# Copper-rich fluids arising from sulfide resorption by hydrous arc melts

**DOI:** 10.1038/s41598-025-29115-x

**Published:** 2026-01-10

**Authors:** Olivia R. Hogg, Marie Edmonds, Penny E Wieser, Matthew Gleeson, Frances E Jenner, Jon Blundy

**Affiliations:** 1 Department of Earth Sciences, University of Cambridge, Cambridge, CB2 3EQ UK; 2 Department of Earth and Planetary Science, UC Berkeley, Berkeley, CA 94720 USA; 3 School of Environment, Earth and Ecosystem Sciences, The Open University, Milton Keynes, MK76AA UK; 4 School of Earth Sciences, University of Oxford, Oxford, OX13AN UK

**Keywords:** Geochemistry, Economic geology

## Abstract

Increasing global demand for copper (Cu) related to the energy transition requires that we understand the mechanisms by which Cu is enriched in the upper crust via magmatism. Porphyry Cu deposits (PCDs) are associated with arc volcanic systems and form under rare circumstances by precipitation from Cu-rich magmatic fluids. Here we develop models to delineate the magmatic conditions under which the Cu concentration and flux may be maximised in exsolved hydrous magmatic fluids. We show that ubiquitous sulfide saturation is a critical limitation on the Cu and sulfur load of exsolved magmatic fluids, owing to the strong partitioning of Cu into sulfide. Sulfide saturation in arc magmas may usually only be avoided under the most hydrous or oxidised conditions, which the global volcanic rock record suggests is not commonplace. However, thermally mature arc crust is likely to develop deep crustal cumulate zones in which sulfides may accumulate over time. When sulfide-undersaturated water-rich mafic melts percolate through these zones they may resorb sulfides during reactive flow. On volatile saturation, Cu-rich fluids will be generated that are viable precursors to PCDs.

## Introduction

The strive towards a carbon-neutral economy comes with increased demand for a number of critical metals, including chalcophile elements such as Cu. Over 70% of global Cu supply is linked to porphyry copper deposits (PCDs) associated with large crustal magmatic intrusions of intermediate composition at convergent margins. Economic enrichments of Cu in the shallow crust are thought to be formed by the precipitation of sulfides from metal-enriched, saline fluids derived from underlying magma bodies in the crust^1^. Mineralisation is influenced by a number of factors such as tectonic regime^2^, fluid focusing^3,4^, long-lived thermal sustainability^5,6^ and precipitation efficiency^7^. However, a critical preliminary step is the generation of magmatic fluids that are rich in both Cu and S; therefore, quantifying how S and Cu-rich fluids form in magmas and under what conditions their concentrations and mass fluxes are optimised may be important in understanding why economically important PCDs are generated above some subduction zones but not others. Understanding the magmatic pathways of Cu through the crust is complex, as chalcophile elements partition strongly into both sulfide and exsolved volatile phases and their partitioning behaviour depends on temperature, pressure, redox state, melt composition and magma volatile content^8,9^.

**Fig. 1:**
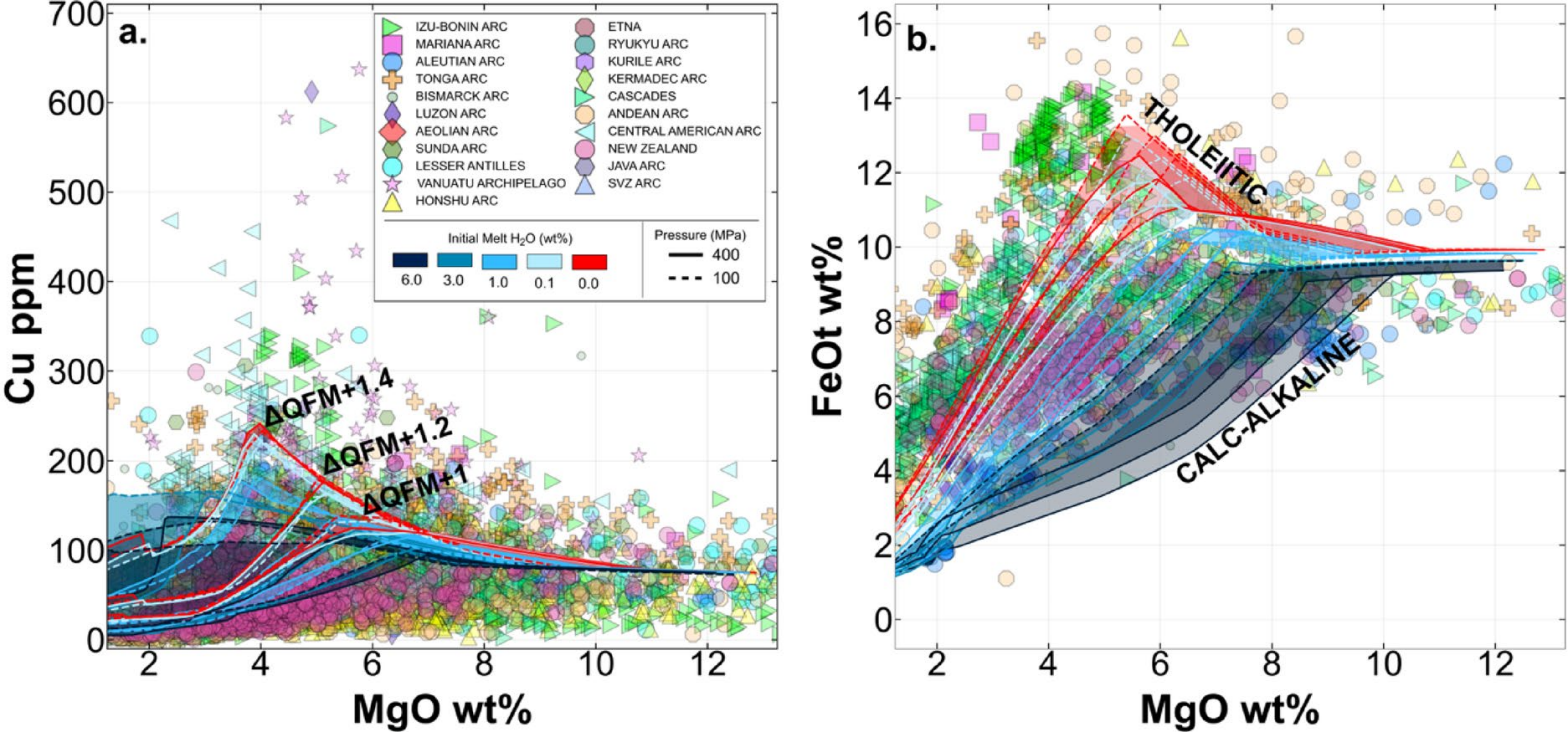
Global whole rock volcanic arc rock compositions (from GEOROC, https://georoc.eu/), showing (a) Cu and (b) FeO_t_ against MgO concentrations. Data are coloured for individual arcs and filtered^15^. Models incorporating crystallisation, sulfide saturation and degassing are shown for different pressures and water concentrations and for ΔQFM + 1, + 1.2 and + 1.4^16^. Initial melt Cu and S concentrations are 75 and 1000 ppm respectively. Water-rich systems (H_2_O > 3 wt%) follow calc-alkaline trends and water-poor systems (H_2_O < 3 wt%) follow tholeiitic trends.

Intermediate arc volcanic rocks (with 3–7 wt% MgO) display a wide range in bulk Cu concentrations from < 50 to > 600 ppm^10^ (Fig. 1a). Giant PCDs require high masses of S and Cu to form. PCDs are often found in continental, convergent-margin settings associated with relatively Fe- and Cu-poor felsic calc-alkaline rocks, which raises questions regarding how and when the Cu was lost from the magma during fractionation and whether this process is important in the mass transfer of these elements to sites of ore formation^11,12^. Primitive magmas (MgO > 7 wt%) from a range of tectonic settings display a limited range in Cu (70–100 ppm)^10^, comparable to that in primitive MORB^13^. Hence, despite porphyries being found exclusively at convergent margins, these magmas do not appear to be predisposed to high Cu concentrations, which hints at the importance of crustal processing for development of their ore-forming potential^14^.

The range in whole rock Cu concentrations in the natural dataset produced during magmatic differentiation (Fig. 1a) may be influenced by crustal thickness^12^, slab thermal parameters^4,12^ or differences in magmatic water concentrations^4,17^. Thick crust may promote high pressure fractionation of garnet, which depletes the melt in FeO^18^; and high magmatic water concentrations promote the high temperature fractionation of Fe-rich phases (e.g., amphibole), resulting in Fe-poor calc-alkaline magmas^15,19^ (Fig. 1b). Decreasing melt FeO content during fractionation lowers the sulfur content at sulfide saturation (SCSS^2−^) of a melt, causing sulfide fractionation and the development of Cu-depleted calc-alkaline melts^11,12,15^. The ‘missing copper’ may be accounted for by deep crustal cumulates^13,20^, suggesting that the mid to lower crustal cumulate zones may act as a ‘trap’ for chalcophile elements such as Cu^13,21^ under certain circumstances.

Volatiles may play an important role in chalcophile element processing, from influencing phase equilibria and the timing and extent of sulfide saturation to providing an exsolved fluid reservoir to transport chalcophile elements. The dissolved water concentrations of primitive arc basalts can vary significantly from < 1 up to > 6 wt%^22,23^. Many workers now consider that super-hydrous melts (with > 10 wt% H_2_O) may be common in arc settings^24,25^. Crystallization during cooling drives magmas to higher water concentrations up until the point of magmatic volatile phase (MVP) saturation, which depends on the confining pressure. Water-rich magmas exsolve a volatile phase at higher melt fractions, higher crustal pressures, and exsolve a far greater mass of fluids relative to water-poor systems during differentiation^17,26^. The concentration of dissolved water in magmas also influences the relative timing and/or depth of degassing and sulfide saturation^27^. Sulfur, Cu and chlorine (Cl) partition strongly into fluids exsolved from oxidised, evolved silicate melts at mid- to upper crustal pressures^28,29^, yet the effect of degassing on the partitioning of S and Cu between melt, sulfide and exsolved fluid phases has not been fully explored.

Previous studies have demonstrated the potential of sulfide transport and resorption for producing Cu-rich magmas and fluids^14,30,31^. Sulfide resorption has been inferred in mineralized^3,14,32^ and unmineralized^31,33^ volcanic systems and is considered by some an essential prerequisite for generating Cu-rich fluids with ore-forming potential^14^. It has been detected cryptically through analysis of chalcophile element ratios (Cu/Ag, Cu/Au) in exsolved hydrous fluids^33,34^, whole rocks^14^ and volcanic glasses^31^ and through S isotope measurements and sulfide grain textures in deep crustal sections^21^. and mafic enclaves^35^. If sulfides are available to interact with aqueous bubbles directly, S and Cu may transfer to the MVP^36^. Interaction between accumulated sulfides in magma reservoirs and cumulate zones^21^ and intruding sulfide-undersaturated melts may be a mechanism to develop Cu-rich aqueous magmatic fluids and melts in settings associated with PCD formation^14^, but the process has not yet been explored quantitatively.

Here we present a novel model that simultaneously describes sulfide saturation and degassing during magma fractionation to establish their effects on the mass distribution of S and Cu among melt, fluid and sulfide reservoirs. We extend this modelling to understand the potential role of sulfide resorption in generating Cu-rich exsolved aqueous magmatic fluids. We investigate the relative importance of sulfide saturation and/or resorption and degassing for sequestration of Cu in evolving melts throughout the crust and establish the optimal conditions for generating the Cu-rich fluids that may play a role in the development of porphyry copper deposits in the shallow crust.

## Results

### Reproducing the global volcanic arc Cu array

**Fig. 2:**
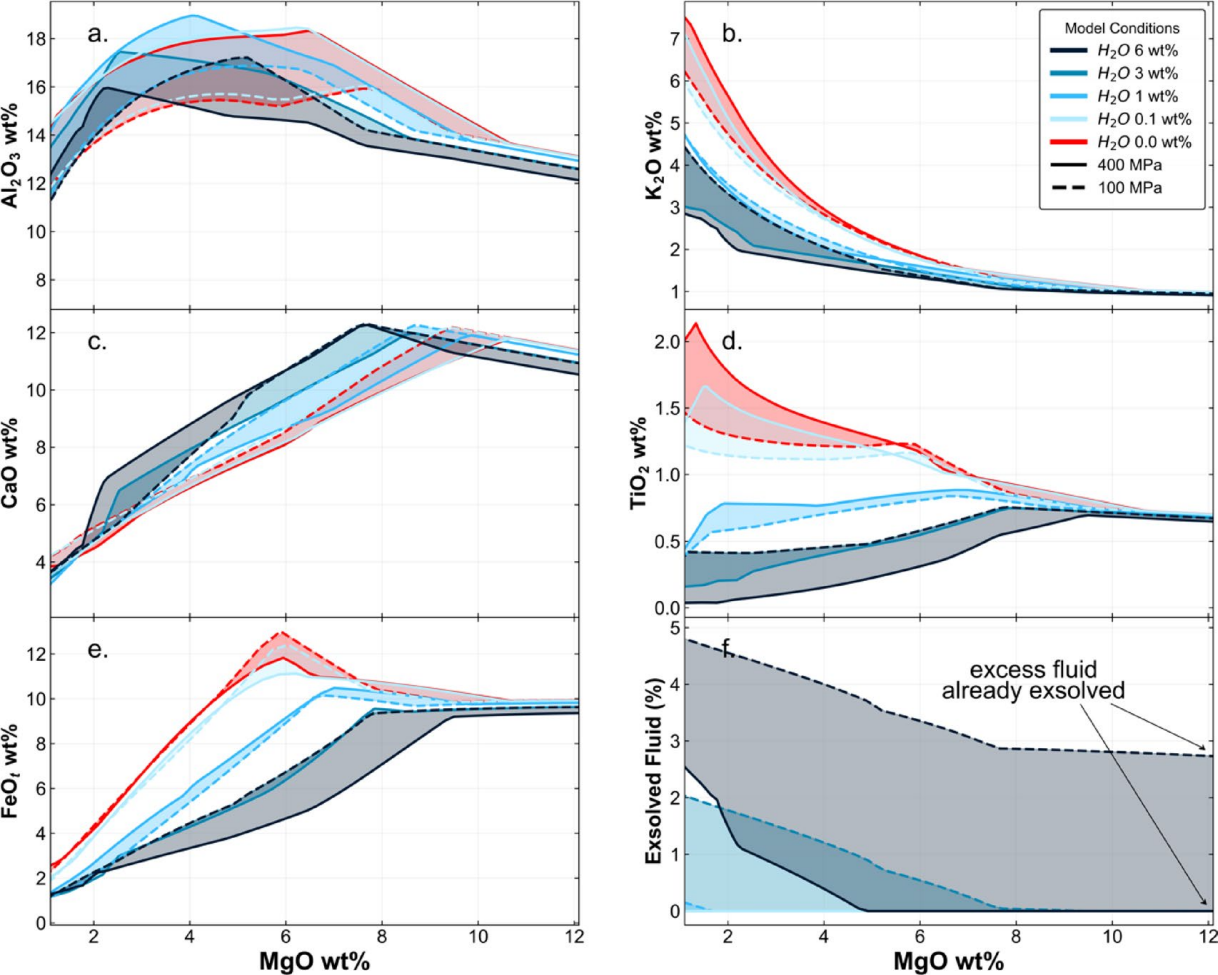
Major element diagrams demonstrating the effect that changing magma water contents has on melt composition during fractional crystallisation (buffered to ΔQFM + 1.2) over a range of pressures (shaded area represents pressures from 100 to 400 MPa), using RhyoliteMELTS^16^.

 Melt compositions arising from isobaric fractionation, degassing and sulfide saturation are shown in Fig. 2. The effect of melt water content is most strongly observed in melt TiO_2_, FeO and K_2_O composition, due to the combined effects of early fractionation of Fe-rich minerals and delayed plagioclase fractionation in wet magmas; and the lower melt fraction for a particular MgO content for wet relative to dry magmas (leading to higher concentrations of incompatible elements such as K_2_O in dry magmas). The development of a significant MVP during fractionation is also of note for magmas containing 1 or more wt% H_2_O. We plot the outputs of the model runs onto the global volcanic arc Cu array in Fig. 1 and it is clear that our model is able to reproduce the first order features of the array despite using a constant initial S and Cu melt content, which is a simplification. It is clear that, as demonstrated previously^17^, higher pressure fractionation of water-rich melts can reproduce the Fe-poor calc-alkaline magma series, whereas lower pressure fractionation of drier magmas produces the tholeiitic magma series.

**Fig. 3:**
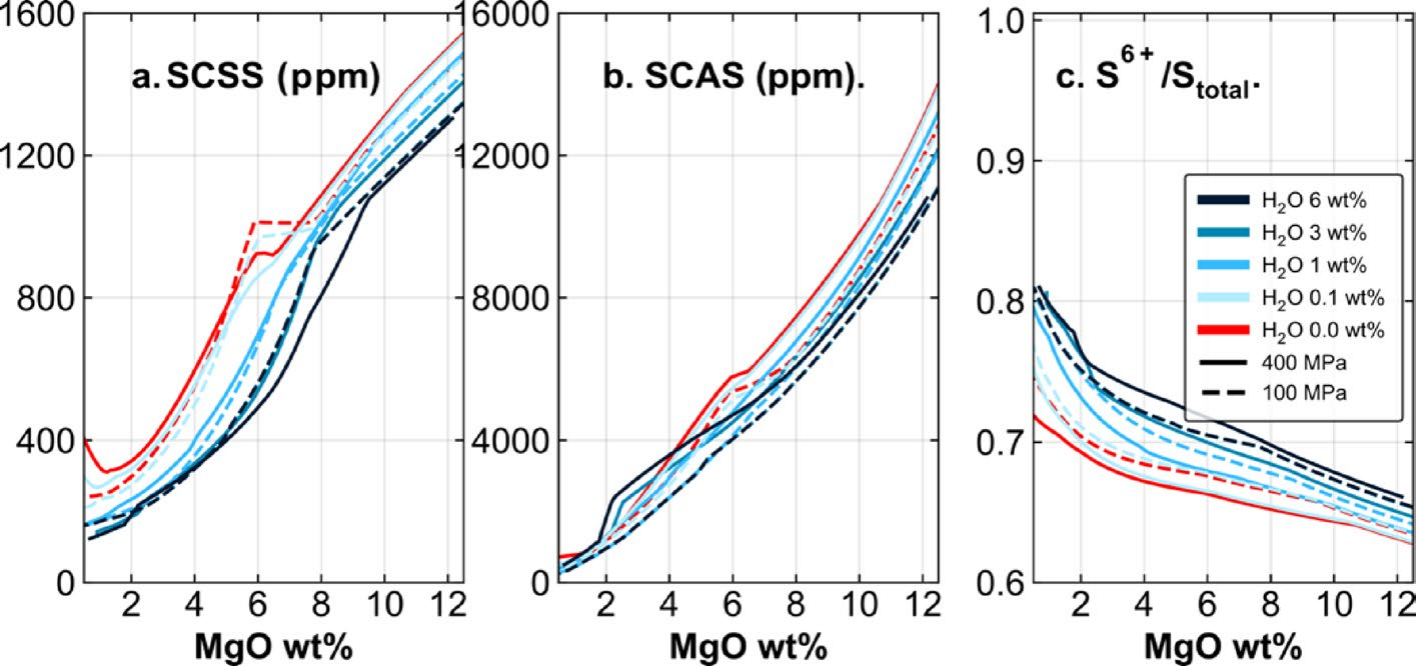
Models of the saturation limit of sulfur-bearing phases in the melts produced by fractional crystallisation over a range of temperature and pressure: (**a**) sulfur concentration at sulfide saturation, SCS^2−^S ; (**b**) sulfur concentration at sulfate saturation, SCAS and (c) the ratio S^6+/^S_total_^37–39^.

The sulfur concentrations at sulfide and sulfate saturation (SCS^2−^S and SCAS) arising from these melt compositions and conditions of pressure and temperature are shown in Fig. 3^37,38^. In general, as expected, the SCS^2−^S decreases dramatically with decreasing melt MgO content and temperature, which promotes ubiquitous sulfide saturation during fractionation, even for relatively oxidised magmas, where the S^6+^ fraction is higher than 50% (Fig. 3C). The SCAS is sufficiently high that magmatic anhydrite is not a significant reservoir for S until the very latest stages of melt evolution and does not sequester Cu, so we do not consider it further.

**Fig. 4:**
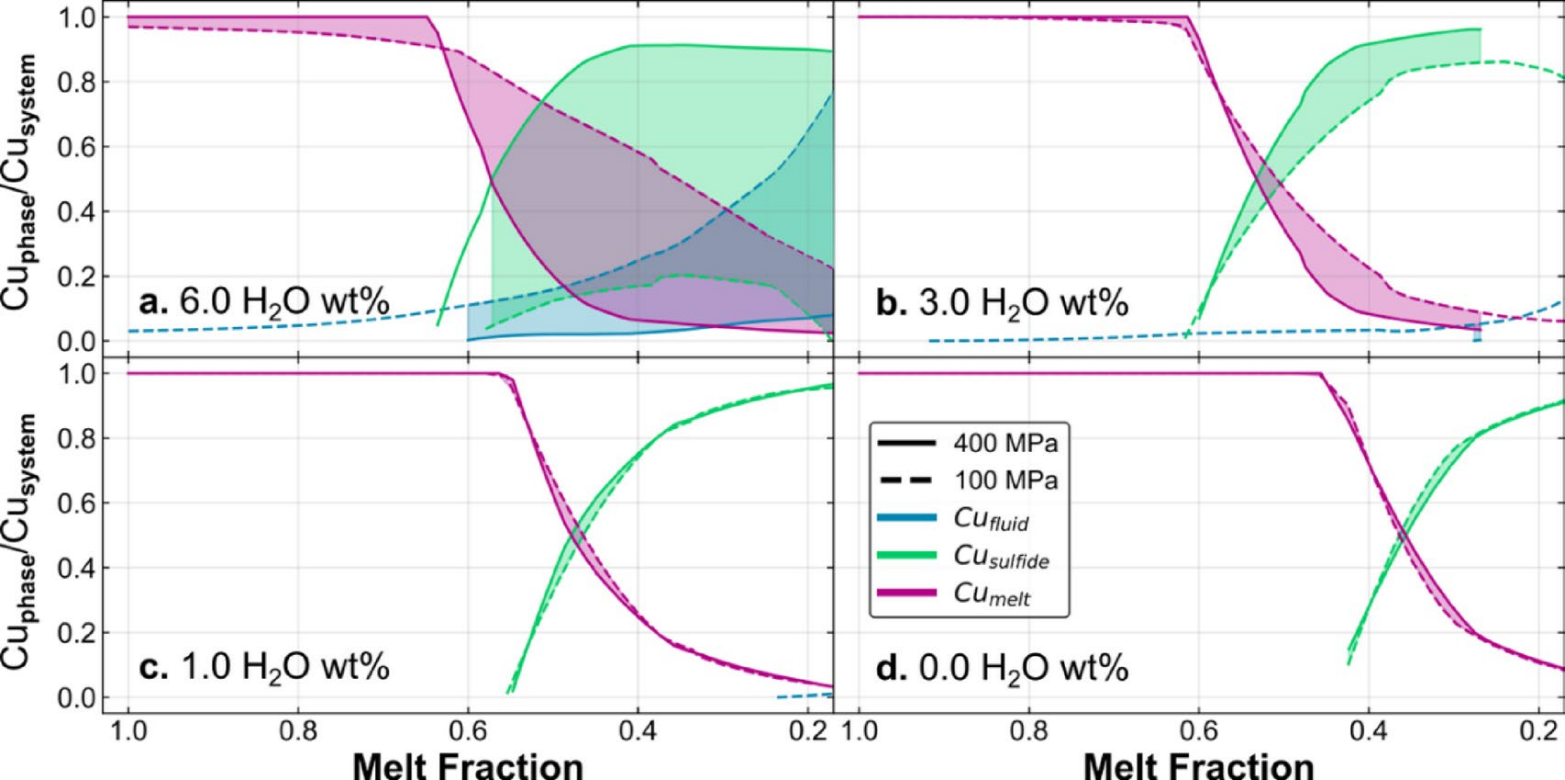
The effect of magma water concentration and pressure on the mass distribution of Cu in the melt-fluid-sulfide system for pressures of 100 and 400 MPa. Cu_phase_ is the mass of Cu in the fluid, melt or sulfide; Cu_system_ is the total mass of Cu in the system (in this case, 1 kg magma). Modelling results for four different magmatic water contents are shown: (**a**) 6 wt%; (**b**) 3 wt%; (**c**) 1 wt%; and (**d**) 0.1 wt% (where no fluid exsolves from the melt).

As the model runs, at each step Cu, S and Cl partition between melt, MVP and sulfide phases, as governed by their respective partition coefficients. Figure 4 shows the relative importance of these three reservoirs for the sequestration of Cu for different magma water compositions. The first aspect to note is that sulfide saturation is ubiquitous and occurs at a higher melt fraction with increasing melt water contents: a melt fraction of 0.65 for melts containing 6 wt% H_2_O versus 0.45 for melts containing 0.1 wt% H_2_O. Water-rich systems (with initially 3–6 wt% H_2_O) experience degassing prior to sulfide saturation (Fig. 4a, b); whereas water-poor systems (0.1–1 wt% H_2_O) become sulfide-saturated before they saturated in an exsolved volatile phase (MVP). For the more water-rich melts the MVP is present virtually along the whole liquid line of descent but does not play a large role in sequestering Cu until melt fractions of 0.1 are reached for the highly water-rich case (Fig. 4a), where the mass of Cu present in the MVP is approximately half that in the sulfide reservoir. In general, however, Cu is efficiently transferred from the melt to the sulfide phase at low melt fractions during isobaric fractionation, regardless of melt water content, leading to a low Cu load in the MVP.

**Fig. 5:**
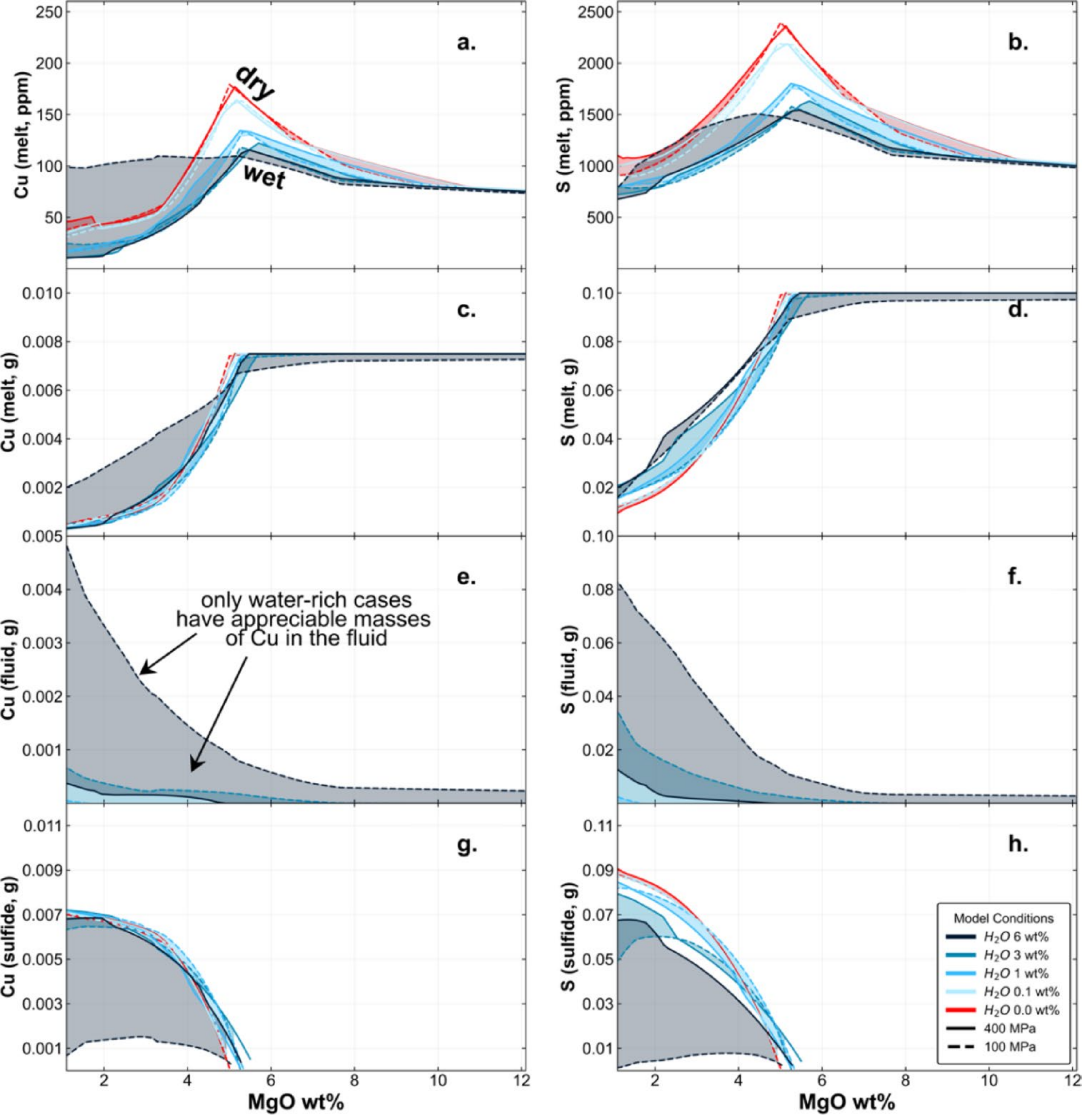
The distribution of Cu and S among melt, sulfide and fluid phases: melt concentrations (**a**, **b**); melt mass (**c**, **d**); fluid mass (**e**, **f**); sulfide mass (**g**, **h**). Dry (water-free) system is highlighted in red and water-bearing systems become progressively darker in blue.

The concentrations of Cu and S in the melt are shown in Figs. 5a and b, which shows that Cu concentrations increase in the melt initially, as the melt fraction decreases during fractionation, and then decrease abruptly at the onset of sulfide saturation. The mass of Cu and S in the melt, in g per kg, is shown in Figs. 5c and d, which eliminates the effect of changing melt fraction. Here we observe the mass of Cu and S in the melt reservoir is constant at high MgO contents until sulfide saturation occurs. Figures 5e-h show the mass of Cu and S in the fluid and sulfide reservoirs. It is clear that while sulfide is present, the Cu and S load of the fluids are generally very low apart from the highly water-rich case, where sulfide formation is suppressed. The MVP is therefore in general not capable of developing high Cu loads for sulfide-saturated magmas in arcs (regardless of MVP salinity), except in exceptionally hydrous or oxidised cases.

### Cu-rich fluids generated by resorption of sulfides

**Fig. 6:**
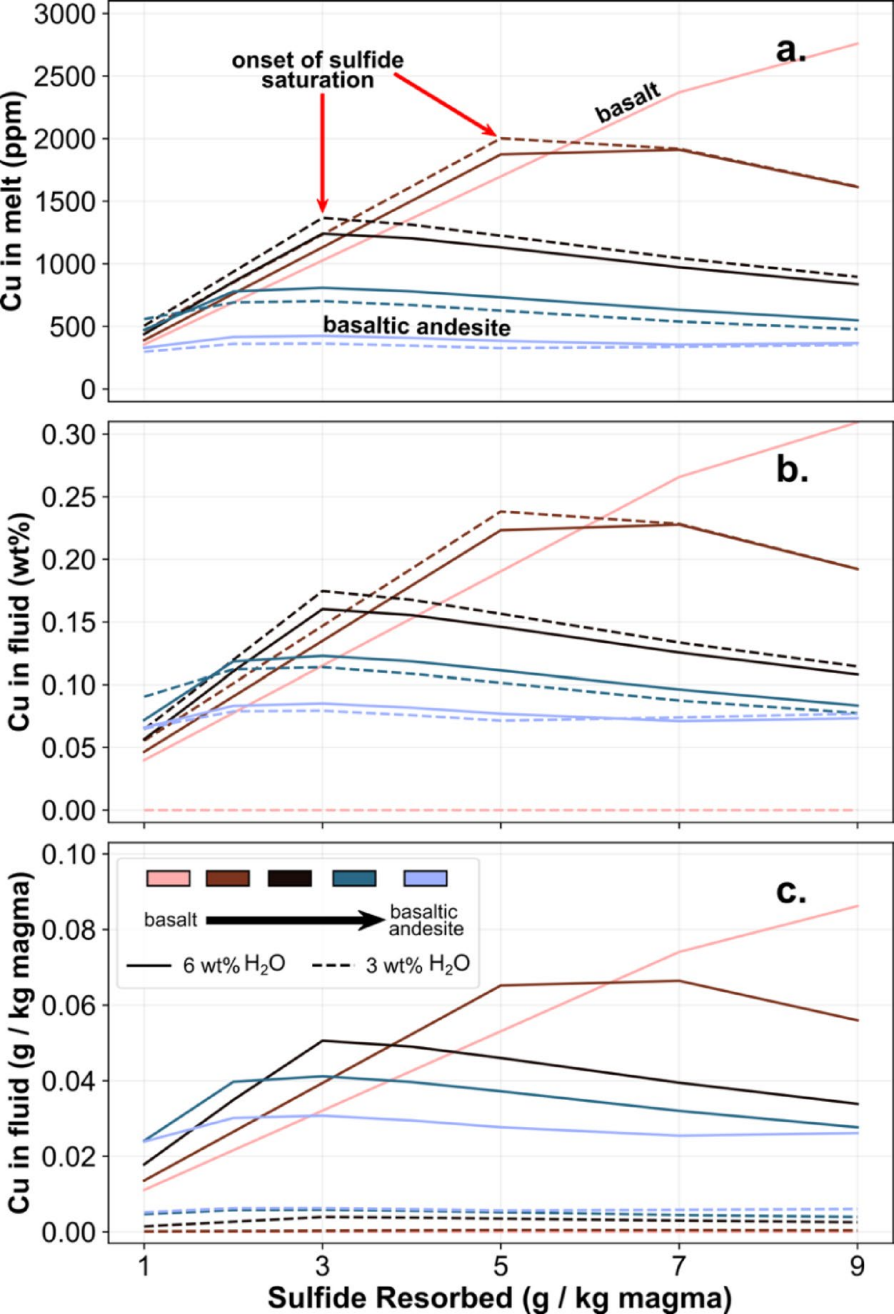
The effect of sulfide resorption on (**a**) melt Cu concentrations (ppm); (**b**) the concentration of Cu in exsolved fluids (wt%) and (**c**) the mass flux of Cu generated via the exsolved fluids from the magma in g of fluid per kg magma. The composition of intruding magma ranges from basalt (melt fraction 1) to basaltic andesite (melt fraction 0.6) and contains 3 and 6 wt% H_2_O. All cases start with melt concentrations of 75 ppm Cu and 1000 ppm S. Basaltic melts can resorb higher masses of sulfide prior to becoming sulfide-saturated compared to basaltic andesitic melts. The most water-rich magmas generate the highest fluxes of Cu-rich fluids.

 Here we model resorption of sulfides (that may be present in a crustal mush) by percolating sulfide-undersaturated melts (Fig. 6). We perform these models for low crustal pressures to illustrate the effect of sulfide resorption on exsolved fluid composition, the mass of which is greatest for low pressure conditions. We show that, for the model conditions here, up to 7 g sulfide may be assimilated per kg magma for the most primitive intruding magmas (i.e. those with the lowest S^2−^ concentrations) before the SCS2-S limit is reached. Resorption of sulfide will increase S and Cu concentrations in both melt and co-existing aqueous magmatic fluid by up to 6 times, depending on the nature and composition of the sulfide phase (Fig. 6b). Water-rich melts that have resorbed sulfides will generate high mass fluxes of Cu in the aqueous exsolved fluid phase (Fig. 6c) simply as a consequence of the high mass of the fluid reservoir, making this a viable mechanism for generating high mass fluxes of Cu-rich fluids in mature arcs where significant regions of sulfide-rich cumulates may have formed.

## Discussion

Our models show that the formation of sulfides is ubiquitous in arc magmas and diminish the ability of the magmatic volatile phase (MVP) to carry Cu (and S) over a large range of conditions of pressure and melt composition due to the strong partitioning of Cu into sulfide. A Cu-barren MVP is therefore expected to form for drier magmas and as the proportion of sulfide decreases towards zero the MVP can become Cu-rich. We therefore expect that the most hydrous (primary melts with ~ 6 wt% or more H_2_O) or most oxidised magmas (whereby the bulk of the sulfur is present as S^6+^) will generate the most Cu-rich MVP, where sulfide saturation may be insignificant or suppressed. An increasing body of work suggests that superhydrous melts (with > 10 wt% H_2_O) may be more common than previously thought^24,25^, their existence perhaps obscured by the resetting of or preferential trapping of melt inclusions in low pressure magma reservoirs, and the fact such melt inclusions are unquenchable (i.e. highly crystallized) so would typically be discarded during sample preparation. We also note that previous work has suggested that excessive water contents may lead to early fluid saturation and loss of fluids at deep crustal levels, which may preclude the formation of shallow crustal porphyry deposits^40^.

Here we use our novel new models to test an alternative mechanism for generating a Cu-rich MVP that might develop in a mature continental arc. It has been proposed that deep crustal mush zones may develop in the arc crust over time^41^. In the early stages of this process, injected mafic sills freeze on short timescales but eventually quasi-stable regions of melt are formed at the top of the injection zone. Over time, the temperature and melt fraction of the mush zone increases to form long-lived mush that may periodically supply shallower reservoirs with relatively evolved buoyant melts that have been formed by reactive flow. Crustal sections are broadly consistent with this view, and it has been shown that significant (negatively-buoyant) sulfide exists in deep crustal cumulate zones which represent the Cu that is missing from the volcanic rock record^,20,21^. The Ivrea crustal section exposes lower crustal cumulates that contain a range of sulfide types^21^. Copper-rich sulfide here exists as an interstitial phase that forms vein networks on a cm scale that has been interpreted to have formed in the temperature range at which Cu-Au (+ Te-Pt-Pd) sulfide is liquid whereas Ni-Fe sulfide is crystalline (as monosulfide solution solution; mss), proposed to be in the region of 1090–1160 °C^21^. It was proposed that such buoyant sulfide liquid may be able to migrate along grain boundaries under conditions of prolonged magmatism whereby melt fractions and permeability are increasing. An interesting feature of this model is the dependence of sulfide liquid mobility on temperature and thus thermal maturity of the magmatic system: if the temperature dips below the Cu-rich sulfide liquid solidus, it will crystallise into an intermediate solid solution (iss). Here we propose, consistent with previous work on the Gangdese suites^14^, that percolating water-rich sulfide-undersaturated silicate melts in such a mature mush system may resorb these interstitial sulfide liquids and/or iss. These S- and Cu-rich silicate melts will saturate in an exsolved volatile phase that will be rich in S and Cu by virtue of their partitioning behaviour, with fluid salinity enhancing fluid-melt partitioning of Cu^29^ and more water-rich melts saturating deeper in the crust and generating larger reservoirs of exsolved fluids. The Cu concentrations of melts and exsolved fluids may be almost an order of magnitude higher than melts and fluids that do not resorb sulfides. High concentrations of PGE elements in Cu +/-Au-bearing granitic suites^42^ compared to barren suites may be consistent with such a model of sulfide resorption via deep crustal cumulates, which may boost the chalcophile element concentrations in melts at the point of volatile saturation^42^.

## Methods

To evaluate the role of increasing primary melt H_2_O concentrations on the partitioning of chalcophile elements between melts, sulfides and fluids, we model isobaric crystallisation, degassing and sulfide saturation in incremental steps starting with a primitive basalt composition (12 wt% MgO)^43^.

### Fractional crystallisation

We model the composition of the melt during isobaric crystallisation and degassing from liquidus temperatures to 800 °C for a range of magma water concentrations (0.1, 1.0, 3.0 and 6.0 wt%), storage pressures (50 MPa to 1 GPa) and oxygen fugacities (ΔQFM + 1 to + 1.2) using RhyoliteMelts^16^ run through the open-source Python3 tool, PetThermoTools^44^. We neglect the presence of carbon dioxide in the melt and we acknowledge we cannot model amphibole fractionation using this method. We have chosen to run the models buffered to a particular oxygen fugacity. We acknowledge that in the natural system there may be changes in *f*O_2_ due to fractionation and degassing, e.g. reduction upon magnetite fractionation. The S and Cu concentrations of the starting basaltic melt were fixed (1000 ppm and 100 ppm, respectively). We assume that Cu is incompatible in all phases aside from sulfide, such that the melt Cu concentration evolves in a simple way with melt fraction. These models therefore produce, for each model run at a fixed pressure, a sequence of melt compositions and the mass fraction of exsolved water for each temperature step. For the most water-rich cases (3–6 wt% H_2_O), a magmatic volatile phase already exists prior to crystallisation and is degassed at the start of our models prior to any crystallization.

### Partitioning of elements into the MVP

**Fig. 7:**
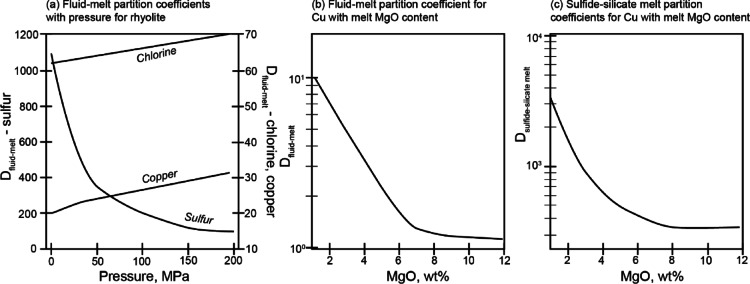
Partitioning behaviour of Cu in the model: (**a**) fluid-melt partition coefficient for sulfur (left axis), chlorine and copper (right axis) with pressure  (**b**) fluid-melt partition coefficient for Cu with melt MgO content and (c) sulfide-silicate melt partition coefficient for Cu with melt MgO content. D_fluid−melt_ for Cu increases proportional to the salinity of the fluid increases during magma differentiation to lower MgO^29^. In **c**) sulfide-silicate melt partition coefficient are parameterised from the literature relevant for sulfide liquid up to FeOt 3.6 wt%^8,53,54^ and MSS^8^ below this threshold.

As the MVP forms during crystallisation, sulfur (S), chlorine (Cl) and Cu partition into it, described by a fluid-melt partition coefficient. Cu is Cl-complexing and its fluid-melt partition coefficient increases with MVP salinity^45,46^ (Fig. 7a). We use parameterised fluid-melt partition coefficients for Cl from^29^, which are 1–3 for basalts (1–3) and 25–65 for rhyolites (25–65) and correlate positively with pressure. Fluid-melt partition coefficients for S are 1–10 for basalts and 15–200 in rhyolites (Fig. 7b) and correlate negatively with pressure^28,47–51^ (Figure 7a). Sulfur fluid-melt partitioning is highly dependent on *f*O_2_^49^. There is evidence that Cl influences the fluid-melt partitioning behaviour for S^52^, but we do not include that effect as it is not sufficiently well constrained. We buffer models run through RhyoliteMELTS at ΔQFM + 1 to 1.2 and therefore use oxidised values for DS_fluid−melt_.

The mass of elements S, Cl and Cu transported by fluids is controlled by the mass of the fluid reservoir, which is a function of magma water content, pressure and the extent of crystallisation. The fluid-melt partition coefficient for element X (S, Cl or Cu) can be written as:

1 $$\:{D}_{fluid-melt}^{X}=\frac{{\left[X\right]}_{fluid}}{{\left[X\right]}_{melt}}=\raisebox{1ex}{$\left(\raisebox{1ex}{${M}_{fluid}^{X}$}\!\left/\:\!\raisebox{-1ex}{${M}_{fluid}^{}$}\right.\right)$}\!\left/\:\!\raisebox{-1ex}{$\left(\raisebox{1ex}{${M}_{melt}^{X}$}\!\left/\:\!\raisebox{-1ex}{${M}_{melt}^{}$}\right.\right)$}\right.$$

Where $\:{D}_{fluid-melt}^{X}$ is the fluid-melt partition coefficient, $\:{\left[X\right]}_{fluid}$ and $\:{\left[X\right]}_{melt}$ are the concentrations of X in the fluid and melt respectively, $\:{M}_{fluid}^{X}$ and $\:{M}_{melt}^{X}$ are the masses of X in the fluid and melt respectively per kg of magma, and $\:{M}_{fluid}^{}$ and $\:{M}_{melt}^{}$ are the masses of the fluid and melt respectively. We calculate the mass of element X in the fluid (for a system mass of magma):

2 $$\:{M}_{fluid}^{X}={M}_{melt}^{X}\times\:\:\frac{{D}_{fluid-melt}^{X}}{G}$$

Where:

$$\:G=\frac{{M}_{melt}^{}}{{M}_{fluid}^{}}$$

Where $\:G$ is the ratio of melt mass to fluid mass in the system.

### Sulfide and sulfate saturation

The relatively oxidised nature of arc magmas means that high proportions of total dissolved S may exist as S^[6 + 39,55^. We use the S speciation model of Jugo et al., (2010) implemented in the open-source Python3 tool PySulfSat^37,38,56^ to calculate melt S^2−^ concentrations from total S. The SCS^2−^S correlates positively with melt FeO and *f*O_2_^37,38^. The model does not take melt water content into account, which has been shown to influence the SCS^2−^S^57,58^.

The fractionation and degassing model described above tracks the concentration of S^2−^ in the melt and, once it exceeds the calculated SCS^2−^S, partitions S^2−^ and Cu into a sulfide liquid. We use sulfide-melt partition coefficients from published studies^8,53^ (Fig. 7c).

The concentration of S^2−^ in the melt, $\:{\left[S\right]}_{melt}^{2-}$ is given by:

3 $$\:{\left[S\right]}_{melt}^{2-}={\left[S\right]}_{melt}^{tot}\times\:\left(\frac{{S}_{melt}^{2-}}{{S}_{melt}^{tot}}\right)$$

Where $\:{\left[S\right]}_{melt}^{tot}$ is the total concentration of S in the melt; and $\:\frac{{S}_{melt}^{2-}}{{S}_{melt}^{tot}}$ is the fraction of S^2−^ in the melt as predicted by speciation models^37^.

Where the $\:{\left[S\right]}_{melt}^{2-}$ is greater than the SCS^2–^S, the concentration and mass of S^2−^ in the sulfide can be calculated as:

4 $$\:{\left[S\right]}_{excess}^{2-}={\left[S\right]}_{melt}^{2-}-{SCSS}^{2-}$$

And the mass of sulfur sequestered in sulfide is:

5 $$\:{M}_{Sulf}^{{S}^{2-}}=\:{M}_{melt}^{tot}\times\:\:{\left[S\right]}_{excess}^{{S}^{2-}}\:$$

where $\:{M}_{melt}^{tot}$ is the total mass of melt. For sulfide-saturated melts, the concentration of sulfate remaining in the melt is equal to the $\:{SCSS}^{2-}$; for sulfide-undersaturated melts, it is equal to$\:{\left[S\right]}_{melt}^{2-}$.

The concentration of sulfate in the melt can be calculated in a similar way:

6 $$\:{\left[S\right]}_{melt}^{6+}={\left[S\right]}_{melt}^{total}\times\:\left(\frac{{S}_{melt}^{6+}}{{S}_{melt}^{Tot}}\right)$$

Where $\:{\left[S\right]}_{melt}^{6+}$ is the concentration of S^6+^ in the melt. Where the $\:{\left[S\right]}_{melt}^{6+}$ is greater than the SCAS, the concentration and mass of S^6+^ in the sulfide can be calculated as:

7 $$\:{\left[S\right]}_{excess}^{6+}={\left[S\right]}_{melt}^{6+}-SCAS$$

And the mass of sulfur sequestered in sulfate (anhydrite) is then:

8 $$\:{M}_{Sulfate}^{{S}^{6+}}=\:{M}_{melt}^{total}\times\:\:{\left[S\right]}_{excess}^{6+}\:$$

where $\:{M}_{melt}^{total}$ is the total mass of melt.

For sulfate-saturated melts, the concentration of sulfate remaining in the melt is equal to the $\:{SCAS}^{}$; for sulfate-undersaturated melts, it is equal to $\:{\left[S\right]}_{melt}^{6+}$.

The total mass of sulfide $\:{M}_{sulfide}^{total}$ may be calculated from the mass of S in the sulfide and the relative molecular mass of S2- relative to other components in the sulfide:

9 $$\:{M}_{sulfide}^{total}={M}_{sulf}^{S2-}\times\:\left[\frac{RM{M}_{sulfide}}{{{RMM}^{S}}_{}}\right]$$

Where $\:RMM$ is the relative molecular mass of the superscripted species. Here we assume a stoichiometry of CuFeS_2_^59^, so this equation simplifies to:

$$\:{M}_{sulfide}^{total}={M}_{sulf}^{S2-}\times\:\frac{63.546\:+55.845\:+2*32.065}{32.065}=\:5.72\:{M}_{sulfide}^{S}$$

While we assume simple stoichiometry for the mass balance above, in reality, sulfides in igneous systems are not CuFeS_2_, but contain appreciable quantities of Ni. While this does not affect the mass significantly given the relatively similar molar masses of Ni and Cu, it does drastically affect the distribution of Cu. Thus, we calculate the mass of Cu sequestered by a sulfide phase ($\:{M}_{sulfide}^{Cu})$ using partition coefficients:

10 $$\:{M}_{sulfide}^{Cu}={M}_{melt}^{Cu}\times\:\:\frac{{D}_{sulfide-melt}^{Cu}}{Y}$$

Where $\:Y$ is the ratio of melt mass to sulfide mass in the system.

Melt concentrations of Cu after the partitioning between melt, fluid and sulfide, $\:{\left[Cu\right]}_{melt\_new}^{}\:$are then calculated using:

11 $$\:{\left[Cu\right]}_{melt\_new}^{}=\frac{\left({M}_{magma}^{Cu}-{M}_{sulfide}^{Cu}-{M}_{fluid}^{Cu}\right)}{{M}_{melt}^{total}}$$

Where $\:{M}_{magma}^{Cu}$ is the total mass of Cu in the system, $\:{M}_{fluid}^{Cu}$ is the mass of Cu partitioned into the MVP (calculated using Eqs. (1 and 2) and $\:{M}_{melt}^{total}$ is the mass of melt at this step.

The new melt Cu concentration was then used to re-calculate fluid and melt distributions of Cu and S. If, after this step, the melt S concentration remained above the SCS^2−^S, Eq. (2 to 11) were iterated to recalculate the total mass of sulfide and the melt S and Cu concentrations until $\:{\left[S\right]}_{melt}^{2-}$ reached a value within 0.1% of the SCS^2–^S.

The model ensures that mass balance in the system is maintained, whereby at the end of each step the check is made that, per kg magma:

12 $$\:{M}_{total}^{X}={M}_{melt}^{X}+{M}_{sulfide}^{X}+{M}_{MVP}^{X}$$

### Sulfide assimilation by percolating melts

 Sulfide-undersaturated magmas ascending through crystal mushes have the potential to assimilate pre-existing sulfides, raising the S and Cu concentration of the melt. We model this process by adding 0.1 g increments of sulfide to mafic to intermediate (F_i_ between 1 and 0.6) sulfide-undersaturated melts with 1000 ppm S and 75 ppm Cu, fractionating at shallow pressures (100 MPa) until the melt became sulfide-saturated, to explore the effect on the evolving fluid composition. The mass of Cu and S added to the melt per 0.1 g increment of sulfide was assumed to have stoichiometry CuFeS_2_. This mass was added to the initial mass of S and Cu in the system and the model was performed again and iterated until the final melt sulfide concentration reached the SCSS^2−^.

## Data Availability

All data generated or analysed during this study are included in this published article.

## Code availability

Code may be found on our GitHub: https://github.com/oliviahogg/CuRichFluid.
